# Supplementary material for: Objectively Measured Sleep Duration and Health-Related Quality of Life in Older Adults with Metabolic Syndrome: A One-Year Longitudinal Analysis of the PREDIMED-Plus Cohort
Source: Nutrients. 2024 Aug 9;16(16):2631. doi: 10.3390/nu16162631 (PMC11357069; doi:10.3390/nu16162631)
Supplement: Supplementary file 1 [file nutrients-16-02631-s001.zip › nutrients-3137751-supplementary.pdf]

SUPPLEMENTARY MATERIAL

Supplementary Figure S1. Flow-chart of study participants.

Supplementary Table S1. Multivariable-adjusted ß-coefficients (95% confidence interval) of physical and mental component summary according to nighttime sleep duration categories, stratified by sex.

Supplementary Table S2. Multivariable-adjusted ß-coefficients (95% confidence interval) of 12-months changes in health-related quality of life according to nighttime sleep duration categories.

Supplementary Table S3. Multivariable-adjusted ß-coefficients (95% confidence interval) of 12-months clinically significative changes in health-related quality of life according to nighttime sleep duration categories.

Supplementary Table S4. Multivariable-adjusted β-coefficients (95% confidence interval) of 12-months clinically significative changes in health-related quality of life according to three daytime sleep duration categories, stratified by nighttime sleep categories.

Supplementary Figure S1. Flow-chart of study participants.

PREDIMED-Plus trial at baseline
n= 6,874

Baseline accelerometry-derived data for this study
n= 2,223

n = 84 participants with incomplete covariates data were excluded

n = 20 participants with incomplete night and daytime sleep data were excluded

Final Sample
n = 2,119
with available SF-36 score and night/daytime data at baseline and 12-months

Supplementary Table S1. Multivariable-adjusted β-coefficients (95% CI) of mental and physical component summary (1 year follow-up) according to nighttime sleep duration categories, stratified by sex

	**Categories of Nighttime Sleep Duration (h/d)**						
	**<6 (n=129)**		**≥6 - <7 (n=316)**		**7-9 (n=1,247)**	**≥9 (n=427)**	
	β-coefficients	*p*-value	*β-coefficients*	*p*-value		*β-coefficients*	*p*-value
				Women (n=1,005)			
**HRQoL, SF-36 Score**							
**PCS**							
Model 1	-5.3 (-8.8 to -1.8)	0.003	-0.4 (-2.2 to 1.4)	0.64	0 (ref.)	1.4 (-2.7 to -0.2)	0.03
Model 2	-4.9 (-8.5 to -1.4)	0.007	-0.6 (-2.4 to 1.2)	0.52	0 (ref.)	-1.5 (-2.7 to -0.2)	0.03
Model 3	-5.1 (-8.6 to -1.5)	0.005	1.0 (-2.8 to 0.8)	0.27	0 (ref.)	-1.2 (-2.5 to 0.06)	0.06
**MCS**							
Model 1	0.6 (-4.5 to 3.4)	0.78	1.6 (-0.5 to 3.6)	0.14	0 (ref.)	-0.8 (-2.2 to 0.7)	0.30
Model 2	1.2 (-2.9 to 5.3)	0.57	1.7 ('-0.4 to 3.7)	0.11	0 (ref.)	0.4 (-1.9 to 1.0)	0.58
Model 3	1.6 (-2.4 to 5.5)	0.43	0.9 (-1.1 to 2.8)	0.40	0 (ref.)	0.1 (-1.3 to 1.6)	0.84
				Men (n=1,114)			
**HRQoL, SF-36 Score**							
**PCS**							
Model 1	-1.6 (-3.1 to -0.08)	0.04	-1.0 (-2.2 to 0.1)	0.09	0 (ref.)	0.9 (-2.1 to 0.4)	0.18
Model 2	-0.9 (-2.5 to 0.7)	0.26	-0.6 (-1.7 to 0.6)	0.33	0 (ref.)	-0.7 (-2.0 to 0.5)	0.25
Model 3	-0.8 (-2.3 to 0.8)	0.34	-0.6 (-1.7 to 0.6)	0.32	0 (ref.)	-0.8 (-2.0 to 0.5)	0.22
**MCS**							
Model 1	0.2 (-1.9 to 1.5)	0.84	0.1 (-1.4 to 1.2)	0.83	0 (ref.)	-0.2 (-1.6 to 1.2)	0.76
Model 2	0.05 (-1.7 to 1.8)	0.95	0.1 (-1.2 to 1.4)	0.88	0 (ref.)	0.09 (-1.5 to 1.3)	0.91
Model 3	0.2 (-1.5 to 2.0)	0.79	0.09 (-1.2 to 1.4)	0.89	0 (ref.)	-0.2 (-1.6 to 1.3)	0.83

Model 1: linear model adjusted for age, marital status (married or living with a partner, divorced or widowed and single), laboral status (active, retirement and unemployed or household work), educational level (≤primary, secondary and university). Model 2: linear model adjusted as in Model 1 and for lifestyle factors: Smoking status (current, former or never smoker), caffeine drinks/day (mg/d), alcohol drinks/day (g/d), leisure-time watching TV (h/wk), adherence Mediterranean diet 17-items questionnaire (continuous), BMI (kg/m2), daytime sleep duration (min/day) and physical recommendations (active/non active). Model 3: linear model adjusted as in Model 2 and for morbidity: Hypertension (yes/no), type-2 diabetes mellitus (yes/no), sedative treatment (yes/no), depression (yes/no), sleep apnea (yes/no), chronic obstructive pulmonary disease (yes/no) and treatment assignment, stratified by sex.

Supplementary Table S2. Multivariable-adjusted ß-coefficients (95% confidence interval) of 12-months changes in health-related quality of life according to nighttime sleep duration categories.

	Categories of Nighttime Sleep Duration (h/d)			
	**<6 h**<br>*n=* 129	**≥6-<7**<br>*n= 316*	**≥7-<9**<br>*n= 1,247*	**≥9**<br>*n= 427*
**HRQoL, SF-36 Score**				
	**Physical Function (PF)**			
**12-month changes,** mean difference (95% CI), points	-1.3 (-5.3 to 2.6)	-2.4 (-4.2 to -0.6)	-4.1 (-5.1 to -3.2)	-3.7 (-5.6 to -1.9)
**Model 1** β-coefficients (95% CI) p-value	0 (ref.)	-0,9 (-4.7 to 2.7)<br>0.61	-2.9 (-6.2 to 0.4)<br>0.08	-2.9 (-6.5 to 0.7)<br>0.11
**Model 2** β-coefficients (95% CI) p-value	0 (ref.)	-1.0 (-4.7 to 2.7)<br>0.61	-2.9 (-6.2 to 0.44)<br>0.09	-2.8 (-6.4 to 0.8)<br>0.13
**Model 3** β-coefficients (95% CI) p-value	0 (ref.)	-1.2 (-4.9 to 2.6)<br>0.55	-3.2 (-6.7 to 1.2)<br>0.06	-3.1 (-6.8 to 0.6)<br>0.10
**Model 4** β-coefficients (95% CI) p-value	0 (ref.)	-0.9 (-4.7 to 2.8)<br>0.62	-2.9 (-6.3 to 0.5)<br>0.09	-2.7 (-6.4 to 0.9)<br>0.14
	**Role Function (RF)**			
**12-month changes,** mean difference (95% CI), points	-6.6 (-14.7 to 1.5)	-4.7 (-8.6 to -0.8)	-5.9 (-8.0 to -3.7)	-6.5 (10.4 to -2.6)
**Model 1** β-coefficients (95% CI) p-value	0 (ref.)	3.0 (-5.1 to 11.1)<br>0.47	2.2 (-5.0 to 9.5)<br>0.54	1.5 (-6.4 to 9.4)<br>0.71
**Model 2** β-coefficients (95% CI) p-value	0 (ref.)	2.9 (-5.2 to 10.9)<br>0.49	2.44 (-4.78 to 9.7)<br>0.51	2.22 (-5.7 to 10.1)<br>0.58
**Model 3** β-coefficients (95% CI) p-value	0 (ref.)	1.5 (-6.7 to 9.7)<br>0.72	0.5 (-7.0 to 8.0)<br>0.90	0.5 (-7.6 to 8.5)<br>0.91
**Model 4** β-coefficients (95% CI) p-value	0 (ref.)	1.5 (-6.8 to 9.7)<br>0.73	0.6 (-6.9 to 8.1)<br>0.88	0.6 (-7.5 to 8.7)<br>0.88
	**Bodily Pain (BP)**			
**12-month changes,** mean difference (95% CI), points	-5.0 (-9.9 to -0.5)	-3.6 (-6.5 to -0.8)	-4.3 (-5.8 to 2.8)	-3.6 (-6.3 to -0.9)
**Model 1** β-coefficients (95% CI) p-value	0 (ref.)	2.0 (-3.6 to 7.5)<br>0.50	1.2 (-3.7 to 6.2)<br>0.62	1.5 (-3.9 to 6.9)<br>0.58
**Model 2** β-coefficients (95% CI) p-value	0 (ref.)	2.0 (-3.6 to 7.5)<br>0.50	1.6 (-3.3 to 6.6)<br>0.52	2.2 (-3.2 to 7.6)<br>0.43
**Model 3** β-coefficients (95% CI) p-value	0 (ref.)	1.7 (-3.9 to 7.3)<br>0.56	1.2 (-3.9 to 6.3)<br>0.64	1.8 (-3.7 to 7.3)<br>0.52
**Model 4** β-coefficients (95% CI) p-value	0 (ref.)	1.8 (3.8 to 7.4)<br>0.53	1.4 (-3.7 to 6.5)<br>0.54	2.1 (-3.4 to 7.6)<br>0.46

	Categories of Nighttime Sleep Duration (h/d)			
	**<6 h**<br>*n=* 129	**≥6-<7**<br>*n= 316*	**≥7-<9**<br>*n= 1,247*	**≥9**<br>*n= 427*
**HRQoL, SF-36 Score**				
	**Vitality (VT)**			
**12-month changes,** mean difference (95% CI), points	-2.4 (-6.0 to 1.3)	-1.9 (-3.9 to 0.02)	-2.1 (-3.2 to -1.1)	-1.1 (-3.0 to 0.8)
**Model 1** β-coefficients (95% CI) p-value	0 (ref.)	1.0 (-3.0 to 4.9)<br>0.63	0.8 (-2.7 to 4.3)<br>0.67	1.6 (-2.3 to 5.4)<br>0.43
**Model 2** β-coefficients (95% CI) p-value	0 (ref.)	0.98 (-3.0 to 4.9)<br>0.63	0.7 (-2.8 to 4.2)<br>0.69	1.5 (-2.3 to 5.4)<br>0.44
**Model 3** β-coefficients (95% CI) p-value	0 (ref.)	0.98 (-3.0 to 5.0)<br>0.63	0.63 (-3.0 to 4.3)<br>0.74	1.4 (-2.5 to 5.3)<br>0.49
**Model 4** β-coefficients (95% CI) p-value	0 (ref.)	1.04 (-2.9 to 5.3)<br>0.61	0.8 (-2.8 to 4.5)<br>0.65	1.7 (-2.2 to 5.7)<br>0.38
	**Social Function (SF)**			
**12-month changes,** mean difference (95% CI), points	0.6 (-5.9 to 3.9)	-0.8 (-3.2 to 1.6)	-0.2 (-1.4 to 1.0)	-0.1 (-2.4 to 2.2)
**Model 1** β-coefficients (95% CI) p-value	0 (ref.)	-0.3 (5.0 to 4.4)<br>0.90	-0.2 (-4.4 to 4.0)<br>0.92	-0.6 (-5.1 to 4.0)<br>0.80
**Model 2** β-coefficients (95% CI) p-value	0 (ref.)	-0.4 (-5.1 to 4.2)<br>0.86	-0.3 (-4.5 to 3.8)<br>0.88	-0.4 (-4.9 to 4.2)<br>0.87
**Model 3** β-coefficients (95% CI) p-value	0 (ref.)	-0.2 (-4.9 to 4.5)<br>0.93	-0.1 (-4.4 to 4.2)<br>0.97	-0.1 (-4.8 to 4.6)<br>0.97
**Model 4** β-coefficients (95% CI) p-value	0 (ref.)	-0.3 (-5.1 to 4.4)<br>0.89	-0.02 (-4.4 to 4.3)<br>0.99	0.1 (-4.5 to 4.8)<br>0.96
	**Emotional Role (RE)**			
**12-month changes,** mean difference (95% CI), points	-4.9 (-11.0 to 1.2)	-2.0 (-5.5 to 1.5)	-3.6 (-5.6 to -1.7)	-3.7 (-7.1 to -0.4)
**Model 1** β-coefficients (95% CI) p-value	0 (ref.)	3.6 (-3.5 to 10.7)<br>0.32	2.3 (-4.0 to 8.6)<br>0.48	2.2 (-4.7 to 9.1)<br>0.54
**Model 2** β-coefficients (95% CI) p-value	0 (ref.)	3.5 (-3.6 to 10.6)<br>0.33	2.2 (-4.2 to 8.5)<br>0.50	2.3 (-4.6 to 9.3)<br>0.51
**Model 3** β-coefficients (95% CI) p-value	0 (ref.)	3.4 (-3.8 to 10.6)<br>0.36	1.8 (-4.8 to 8.4)<br>0.59	2.1 (-5.0 to 9.1)<br>0.57
**Model 4** β-coefficients (95% CI) p-value	0 (ref.)	2.9 (-4.3 to 10.1)<br>0.43	1.7 (-4.9 to 8.3)<br>0.61	2.3 (-4.7 to 9.4)<br>0.52

	**General Health (GH)**			
**12-month changes,** mean difference (95% CI), points	-2.5 (-5.7 to 0.7)	-2.7 (-4.5 to -0.9)	-4.7 (-5.7 to -3.7)	-2.9 (-4.5 to -1.3)
**Model 1** β-coefficients (95% CI) p-value	0 (ref.)	0.1 (-3.5 to 3.6) 0.96	-2.3 (-5.5 to 0.8) 0.15	-1.2 (-4.6 to 2.3) 0.51
**Model 2** β-coefficients (95% CI) p-value	0 (ref.)	0.1 (-2.5 to 3.6) 0.96	-2.2 (-5.4 to 1.0) 0.18	-1.0 (-4.5 to 2.5) 0.58
**Model 3** β-coefficients (95% CI) p-value	0 (ref.)	-0.2 (-3.9 to 3.4) 0.89	-2.7 (-6.0 to 0.6) 0.11	-1.4 (-4.9 to 2.2) 0.45
**Model 4** β-coefficients (95% CI) p-value	0 (ref.)	-0.02 (-3.6 to 3.6) 0.99	-2.5 (-5.7 to 0.8) 0.14	-1.2 (-4.7 to 2.4) 0.52
	**Aggregated Physical Dimensions (PCS)**			
**12-month changes,** mean difference (95% CI), points	-1.2 (-3.0 to 0.5)	-1.5 (-2.4 to -0.6)	-2.0 (-2.5 to -1.6)	-1.7 (-2.6 to -0.8)
**Model 1** β-coefficients (95% CI) p-value	0 (ref.)	-0.1 (-1.8 to 1.7) 0.96	-0.7 (-2.3 to 0.9) 0.39	-0.5 (-2.3 to 1.2) 0.54
**Model 2** β-coefficients (95% CI) p-value	0 (ref.)	-0.1 (-1.8 to 1.7) 0.95	-0.6 (-2.2 to 1.0) 0.46	-0.4 (-2.1 to 1.4) 0.68
**Model 3** β-coefficients (95% CI) p-value	0 (ref.)	-0.3 (-2.1 to 1.5) 0.75	-0.9 (-2.6 to 0.7) 0.27	-0.6 (-2.4 to 1.1) 0.47
**Model 4** β-coefficients (95% CI) p-value	0 (ref.)	-0.2 (-2.0 to 1.6) 0.84	-0.8 (-2.4 to 0.8) 0.33	-0.6 (-2.3 to 1.2) 0.54

	**Mental Health (MH)**			
**12-month changes,** mean difference (95% CI), points	-2.0 (-5.2 to 1.3)	-0.7 (-2.5 to 1.0)	-1.2 (-2.2 to -0.2)	-1.1 (-2.9 to 0.7)
**Model 1** β-coefficients (95% CI) p-value	0 (ref.)	1.3 (-2.3 to 5.0) 0.47	0.7 (-2.5 to 4.0) 0.66	0.6 (-3.0 to 4.1) 0.75
**Model 2** β-coefficients (95% CI) p-value	0 (ref.)	1.3 (-2.3 to 5.0) 0.47	0.7 (-2.5 to 4.0) 0.66	0.6 (-3.0 to 4.1) 0.75
**Model 3** β-coefficients (95% CI) p-value	0 (ref.)	1.4 (-2.3 to 5.1) 0.47	0.7 (-2.7 to 4.1) 0.69	0.5 (-3.1 to 4.1) 0.78
**Model 4** β-coefficients (95% CI) p-value	0 (ref.)	1.4 (-2.3 to 5.1) 0.45	0.8 (-2.5 to 4.2) 0.63	0.8 (-2.8 to 4.4) 0.67
	**Aggregated Mental Dimensions (MCS)**			
**12-month changes,** mean difference (95% CI), points	-0.9 (-2.9 to 1.0)	-0.1 (-1.2 to 1.0)	-0.2 (-0.8 to 0.4)	-0.2 (-1.2 to 0.9)
**Model 1** β-coefficients (95% CI) p-value	0 (ref.)	0.9 (-1.3 to 3.1) 0.42	0.8 (-1.1 to 2.8) 0.41	0.8 (-1.3 to 2.9) 0.47
**Model 2** β-coefficients (95% CI) p-value	0 (ref.)	0.9 (-1.3 to 3.0) 0.44	0.7 (-1.2 to 2.7) 0.45	0.8 (-1.4 to 2.9) 0.49
**Model 3** β-coefficients (95% CI) p-value	0 (ref.)	1.0 (-1.2 to 3.2) 0.38	0.8 (-1.2 to 2.9) 0.41	0.8 (-1.3 to 3.0) 0.45
**Model 4** β-coefficients (95% CI) p-value	0 (ref.)	0.9 (-1.3 to 3.1) 0.44	0.8 (-1.2 to 2.9) 0.41	1.0 (-1.2 to 3.1) 0.39

Model 1: linear model adjusted for age and sex. Model 2: linear model adjusted as in Model 1 and for marital status (married or living with a partner, divorced or widowed and single), laboral status (active, retirement and unemployed or household work), educational level (≤primary, secondary and university). Model 3: linear model adjusted as in Model 2 and for lifestyle factors: Smoking status (current, former or never smoker), caffeine drinks/day (mg/d), alcohol drinks/day (g/d), leisure-time watching TV (h/wk), adherence Mediterranean diet 17-items questionnaire (continuous), bmi (kg/m2), mvpa recommendations (active/non active), and napping (min/d). Model 4: linear model adjusted as in Model 3 and for morbidity: Hypertension (yes/no), type-2 diabetes mellitus (yes/no), sedative treatment (yes/no), depression (yes/no), sleep apnea (yes/no), chronic obstructive pulmonary disease (yes/no) and treatment assignment.

Supplementary Table S3. Multivariable-adjusted odd ratio (95% confidence interval) of 12-months clinical significative changes in health-related quality of life according to nighttime sleep duration categories.

	Categories of Nighttime Sleep Duration (h/d)			
	**<6h**<br>*n= 129*	**≥6-<7 h**<br>*n= 316*	**7-9h**<br>*n= 1,247*	**≥9 h**<br>*n= 427*
**HRQoL, SF-36 Score**				
**Physical Function (PF)**				
**Model 1** Odd Ratio (95% CI) p-value	0.92 (0.63-1.32)<br>0.64	0.80 (0.62-1.02)<br>0.07	0 (ref.)	1.01 (0.81-1.26)<br>0.91
**Model 2** Odd Ratio (95% CI) *P*-value	0.92 (0.64-1.33)<br>0.66	0.80 (0.63-1.03)<br>0.09	0 (ref.)	1.01 (0.80-1.26)<br>0.96
**Model 3** Odd Ratio (95% CI) *P*-value	0.86 (0.59-1.26)<br>0.44	0.79 (0.61-1.01)<br>0.06	0 (ref.)	0.99 (0.79-1.24)<br>0.92
**Model 4** Odd Ratio (95% CI) *P*-value	0.89 (0.61-1.31)<br>0.55	0.78 (0.61-1.01)<br>0.06	0 (ref.)	0.99 (0.79-1.24)<br>0.93
**Role Function (RF)**				
**Model 1** Odd Ratio (95% CI) *P*-value	1.70 (1.13-2.55)<br>0.01	1.01 (0.75-1.36)<br>0.93	0 (ref.)	1.02 (0.79-1.32)<br>0.88
**Model 2** Odd Ratio (95% CI) *P*-value	1.74 (1.16-2.61)<br>0.01	1.03 (0.77-1.39)<br>0.83	0 (ref.)	1.01 (0.78-1.31)<br>0.93
**Model 3** Odd Ratio (95% CI) *P*-value	1.51 (0.99-2.31)<br>0.06	0.98 (0.72-1.32)<br>0.89	0 (ref.)	1.00 (0.77-1.29)<br>0.97
**Model 4** Odd Ratio (95% CI) *P*-value	1.51 (0.99-2.31)<br>0.06	0.99 (0.73-1.34)<br>0.97	0 (ref.)	0.97 (0.75-1.27)<br>0.84
**Bodily Pain (BP)**				
**Model 1** Odd Ratio (95% CI) *P*-value	1.46 (1.01-2.11)<br>0.04	1.07 (0.83-1.38)<br>0.60	0 (ref.)	1.05 (0.84-1.31)<br>0.67
**Model 2** Odd Ratio (95% CI) *P*-value	1.52 (1.05-2.21)<br>0.03	1.11 (0.86-1.43)<br>0.42	0 (ref.)	1.03 (0.83-.1.30)<br>0.77
**Model 3** Odd Ratio (95% CI) *P*-value	1.46 (1.00-2.15)<br>0.05	1.10 (0.85-1.42)<br>0.47	0 (ref.)	1.03 (0.82-1.29)<br>0.82
**Model 4** Odd Ratio (95% CI) *P*-value	1.49 (1.01-2.20)<br>0.04	1.11 (0.86-1.43)<br>0.44	0 (ref.)	1.01 (0.81-1.28)<br>0.91
**General Health (GH)**				
**Model 1** Odd Ratio (95% CI) *P*-value	0.76 (0.53-1.10)<br>0.15	0.83 (0.65-1.06)<br>0.14	0 (ref.)	0.90 (0.72-1.13)<br>0.36
**Model 2** Odd Ratio (95% CI) *P*-value	0.77 (0.53-1.11)<br>0.15	0.90 (0.72-1.12)<br>0.34	0 (ref.)	0.90 (0.72-1.12)<br>0.34
**Model 3** Odd Ratio (95% CI) *P*-value	0.73 (0.49-1.07)<br>0.11	0.82 (0.64-1.06)<br>0.13	0 (ref.)	0.89(0.71-1.11)<br>0.30
**Model 4** Odd Ratio (95% CI) *P*-value	0.74 (0.50-1.09)<br>0.12	0.82 (0.64-1.06)<br>0.13	0 (ref.)	0.88 (0.70-1.11)<br>0.28

	Categories of Nighttime Sleep Duration (h/d)			
	**<6h**<br>*n= 129*	**≥6-<7 h**<br>*n= 316*	**7-9h**<br>*n= 1,247*	**≥9 h**<br>*n= 427*
**HRQoL, SF-36 Score**				
**Vitality (VT)**				
**Model 1** Odd Ratio (95% CI) *P*-value	1.28 (0.89-1.86)<br>0.18	1.08 (0.84-1.38)<br>0.57	0 (ref.)	0.89 (0.71-1.11)<br>0.32
**Model 2** Odd Ratio (95% CI) *P*-value	1.20 (0.90-1.88)<br>0.16	1.08 (0.84-1.39)<br>0.54	0 (ref.)	0.90 (0.72-1.12)<br>0.35
**Model 3** Odd Ratio (95% CI) *P*-value	1.28 (0.87-1.87)<br>0.21	1.06 (0.82-1.37)<br>0.64	0 (ref.)	0.90 (0.71-1.13)<br>0.35
**Model 4** Odd Ratio (95% CI) *P*-value	1.31 (0.89-1.93)<br>0.22	1.08 (0.84-1.40)<br>0.54	0 (ref.)	0.89 (0.71-1.12)<br>0.31
**Social Function (SF)**				
**Model 1** Odd Ratio (95% CI) *P*-value	1.05 (0.68-1.60)<br>0.83	1.04 (0.78-1.38)<br>0.78	0 (ref.)	1.09 (0.85-1.40)<br>0.50
**Model 2** Odd Ratio (95% CI) *P*-value	1.06 (0.69-1.62)<br>0.80	1.05 (0.79-1.40)<br>0.72	0 (ref.)	1.07 (0.83-1.37)<br>0.61
**Model 3** Odd Ratio (95% CI) *P*-value	1.02 (0.65-1.59)<br>0.93	1.03 (0.77-1.37)<br>0.86	0 (ref.)	1.07 (0.83-1.37)<br>0.62
**Model 4** Odd Ratio (95% CI) *P*-value	1.01 (0.65-1.59)<br>0.95	1.07 (0.80-1.43)<br>0.66	0 (ref.)	1.02 (0.79-1.32)<br>0.90
**Emocional Role (RE)**				
**Model 1** Odd Ratio (95% CI) *P*-value	1.15 (0.65-2.01)<br>0.63	1.03 (0.71-1.50)<br>0.86	0 (ref.)	0.96 (0.70-1.33)<br>0.82
**Model 2** Odd Ratio (95% CI) *P*-value	1.16 (0.66-2.03)<br>0.61	1.05 (0.72-1.53)<br>0.79	0 (ref.)	0.94 (0.68-1.30)<br>0.70
**Model 3** Odd Ratio (95% CI) *P*-value	1.03 (0.69-1.46)<br>0.91	1.00 (0.69-1.46)<br>0.99	0 (ref.)	0.92 (0.66-1.28)<br>0.63
**Model 4** Odd Ratio (95% CI) *P*-value	1.01 (0.55-1.83)<br>0.98	1.09 (0.74-1.60)<br>0.66	0 (ref.)	0.83 (0.60-1.17)<br>0.29
**Mental Health (MH)**				
**Model 1** Odd Ratio (95% CI) *P*-value	1.07 (0.73-1.58)<br>0.73	0.84 (0.64-1.10)<br>0.20	0 (ref.)	1.07 (0.85-1.36)<br>0.54
**Model 2** Odd Ratio (95% CI) *P*-value	1.08 (0.73-1.59)<br>0.72	0.84 (0.64-1.11)<br>0.22	0 (ref.)	1.06 (0.84-1.34)<br>0.61
**Model 3** Odd Ratio (95% CI) *P*-value	1.02 (0.68-1.53)<br>0.92	0.83 (0.63-1.09)<br>0.17	0 (ref.)	1.05 (0.83-1.33)<br>0.70
**Model 4** Odd Ratio (95% CI) *P*-value	1.04 (0.69-1.56)<br>0.87	0.83 (0.63-1.10)<br>0.20	0 (ref.)	1.02 (0.80-1.30)<br>0.88

	Aggregated Physical Dimensions (PCS)					Aggregated Mental Dimensions (MCS)			
**Model 1** Odd Ratio (95% CI) *P*-value	0.91 (0.62-1.31)<br>0.60	0.86 (0.67-1.11)<br>0.24	0 (ref.)	0.89 (0.71-1.12)<br>0.33	**Model 1** Odd Ratio (95% CI) *P*-value	1.17 (0.79-1.73)<br>0.42	1.02 (0.78-1.34)<br>0.86	0 (ref.)	0.92 (0.72-1.17)<br>0.48
**Model 2** Odd Ratio (95% CI) *P*-value	0.92 (0.63-1.34)<br>0.66	0.88 (0.68-1.13)<br>0.31	0 (ref.)	0.87 (0.70-1.10)<br>0.25	**Model 2** Odd Ratio (95% CI) *P*-value	1.17 (0.79-1.73)<br>0.44	1.02 (0.78-1.34)<br>0.89	0 (ref.)	0.91 (0.71-1.16)<br>0.44
**Model 3** Odd Ratio (95% CI) *P*-value	0.85 (0.58-1.26)<br>0.43	0.86 (0.67-1.11)<br>0.25	0 (ref.)	0.87 (0.89-1.09)<br>0.22	**Model 3** Odd Ratio (95% CI) *P*-value	1.09 (0.72-1.65)<br>0.68	0.99 (0.75-1.30)<br>0.92	0 (ref.)	0.89 (0.69-1.13)<br>0.33
**Model 4** Odd Ratio (95% CI) *P*-value	0.88 (0.59-1.29)<br>0.51	0.87 (0.67-1.12)<br>0.27	0 (ref.)	0.86 (0.68-1.09)<br>0.21	**Model 4** Odd Ratio (95% CI) *P*-value	1.08 (0.72-1.64)<br>0.71	1.01 (0.77-1.34)<br>0.92	0 (ref.)	0.85 (0.66-1.09)<br>0.19

Model 1: linear model adjusted for age and sex. Model 2: linear model adjusted as in Model 1 and for marital status (married or living with a partner, divorced or widowed and single), laboral status (active, retirement and unemployed or household work), educational level (≤primary, secondary and university). Model 3: linear model adjusted as in Model 2 and for lifestyle factors: Smoking status (current, former or never smoker), caffeine drinks/day (mg/d), alcohol drinks/day (g/d), leisure-time watching TV (h/wk), adherence Mediterranean diet 17-items questionnaire (continuous), bmi (kg/m2), mvpa recommendations (active/non active), and napping (min/d). Model 4: linear model adjusted as in Model 3 and for morbidity: Hypertension (yes/no), type-2 diabetes mellitus (yes/no), sedative treatment (yes/no), depression (yes/no), sleep apnea (yes/no), chronic obstructive pulmonary disease (yes/no) and treatment assignment.

Supplementary Table S4. Multivariable-adjusted odd ratio (95% confidence interval) of 12-months clinically significative changes in health-related quality of life according to three daytime sleep duration categories, stratified by nighttime sleep categories.

	Categories of Daytime Sleep Duration (min/d)				
	**<15** *(n=108)*	**≥15 to <60** *(n=914)*		**≥60** *(n=1,097)*	
		β-coefficients	*p*-value	*β-coefficients*	*p*-value
		Night-time Sleep Duration <7 h/d (n=445)			
**HRQoL, SF-36 Score**					
**Aggregated Physical Dimensions (PCS)**					
Model 1	0 (ref.)	0.58 (0.18-1.91)	0.38	0.84 (0.26-2.71)	0.77
Model 2	0 (ref.)	0.64 (0.19-2.19)	0.84	0.88 (0.26-2.97)	0.84
Model 3	0 (ref.)	0.50 (0.14-1.81)	0.29	0.68 (0.19-2.43)	0.56
Model 4	0 (ref.)	0.48 (0.13-1.76)	0.27	0.73 (0.20-2.61)	0.63
**Aggregated Mental Dimensions (MCS)**					
Model 1	0 (ref.)	0.69 (0.21-2.30)	0.54	0.66 (0.20-2.18)	0.50
Model 2	0 (ref.)	0.69 (0.20-2.40)	0.56	0.62 (0.18-2.12)	0.45
Model 3	0 (ref.)	0.64 (0.18-2.30)	0.50	0.59 (0.17-2.07)	0.41
Model 4	0 (ref.)	0.64 (0.17-2.39)	0.51	0.54 (0.15-1.97)	0.35
		Night-time Sleep Duration ≥7 to <9 h/d (n=1,247)			
**HRQoL, SF-36 Score**					
**Aggregated Physical Dimensions (PCS)**					
Model 1	0 (ref.)	0.65 (0.41-1.05)	0.08	0.83 (0.52-1.34)	0.44
Model 2	0 (ref.)	0.66 (0.41-1.97)	0.09	0.85 (0.52-1.37)	0.50
Model 3	0 (ref.)	0.67 (0.41-1.08)	0.10	0.88 (0.54-1.43)	0.60
Model 4	0 (ref.)	0.66 (0.40-1.07)	0.09	0.87 (0.53-1.42)	0.57
**Aggregated Mental Dimensions (MCS)**					
Model 1	0 (ref.)	1.13 (0.68-1.88)	0.64	1.21 (0.72-2.03)	0.47
Model 2	0 (ref.)	1.13 (0.67-1.89)	0.65	1.20 (0.72-2.02)	0.49
Model 3	0 (ref.)	0.97 (0.58-1.65)	0.92	0.99 (0.58-1.68)	0.96
Model 4	0 (ref.)	1.00 (0.59-1.71)	0.99	0.98 (0.57-1.68)	0.93
		Night-time Sleep Duration ≥9 h/d (n= 427)			
**HRQoL, SF-36 Score**					
**Aggregated Physical Dimensions (PCS)**					
Model 1	0 (ref.)	0.39 (0.13-1.11)	0.08	0.41 (0.15-1.17)	0.10
Model 2	0 (ref.)	0.40 (0.14-1.18)	0.10	0.43 (0.15-1.26)	0.13
Model 3	0 (ref.)	0.33 (0.11-1.01)	0.05	0.33 (0.11-1.04)	0.06
Model 4	0 (ref.)	0.32 (0.10-1.02)	0.05	0.36 (0.11-1.16)	0.09
**Aggregated Mental Dimensions (MCS)**					
Model 1	0 (ref.)	1.23 (0.41-3.68)	0.71	0.98 (0.33-2.91)	0.98
Model 2	0 (ref.)	1.33 (0.44-4.03)	0.62	1.04 (0.35-3.16)	0.94
Model 3	0 (ref.)	1.26 (0.41-3.89)	0.69	0.98 (0.31-3.04)	0.97
Model 4	0 (ref.)	1.26 (0.40-3.97)	0.69	0.95 (0.30-3.01)	0.93

Model 1: linear model adjusted for age, marital status (married or living with a partner, divorced or widowed and single), laboral status (active, retirement and unemployed or household work), educational level (£primary, secondary and university). Model 2: linear model adjusted as in Model 1 and for lifestyle factors: Smoking status (current, former or never smoker), caffeine drinks/day (mg/d), alcohol drinks/day (g/d), leisure-time watching TV (h/wk), adherence Mediterranean diet 17-items questionnaire (continuous), BMI (kg/m2), daytime sleep duration (min/d) and physical recommendations (active/non active). Model 3: linear model adjusted as in Model 2 and for morbidity: Hypertension (yes/no), type-2 diabetes mellitus (yes/no), sedative treatment (yes/no), depression (yes/no), sleep apnea (yes/no), chronic obstructive pulmonary disease (yes/no), stratified by sex.
